# Supplementary material for: Different Involvement of Vimentin during Invasion by Listeria monocytogenes at the Blood–Brain and the Blood–Cerebrospinal Fluid Barriers In Vitro
Source: Int J Mol Sci. 2022 Oct 26;23(21):12908. doi: 10.3390/ijms232112908 (PMC9658511; doi:10.3390/ijms232112908)
Supplement: Supplementary file 1 [file ijms-23-12908-s001.zip › ijms-1968232-supplementary.pdf]

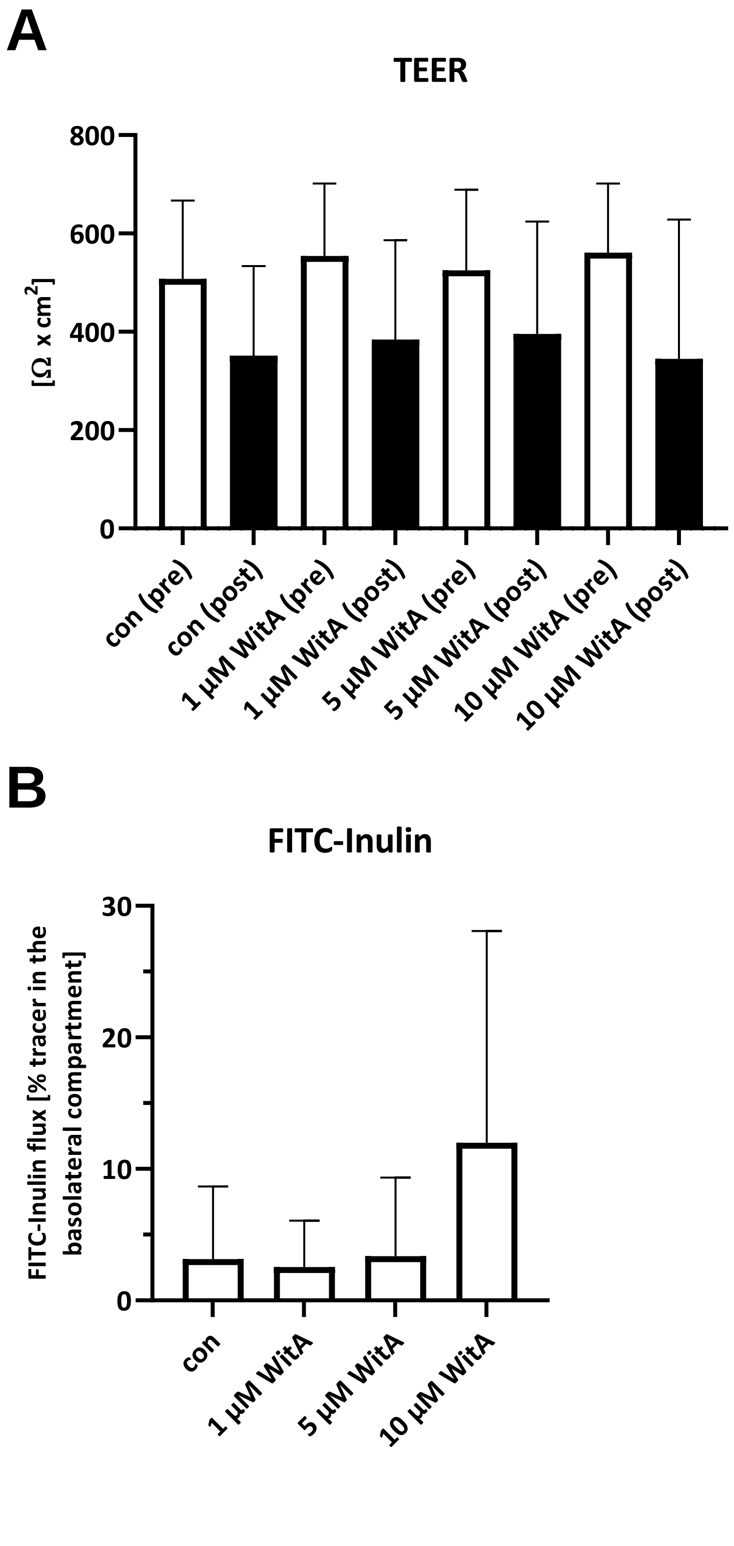

Supplementary Figure S1. HIBCPP cells grown in the inverted cell culture system were pre-incubated in absence (con) and in presence of the indicated concentrations of WitA, and subsequently infected with wild type *Lm* EGD-e (MOI = 10) and co-incubated for 4 h. (A) TEER values were measured at the beginning (pre) and the end (post) of the experiment. (B) The flux of FITC-labelled inulin (FITC-Inulin) was determined at the end of the experiment. Shown are the results of three independent experiments performed in triplicates.

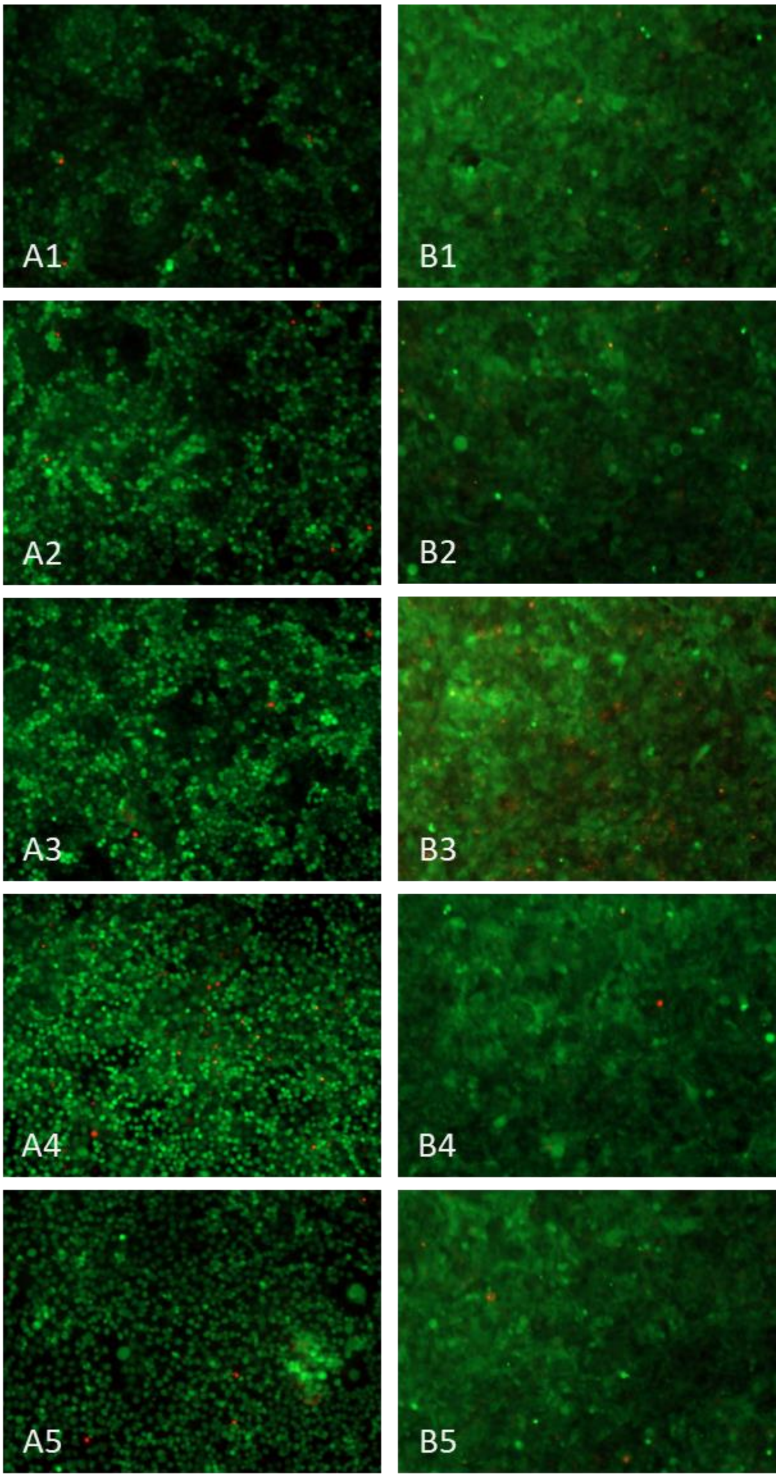

Supplementary Figure S2. Co-incubation with WitA in presence of *Lm* does not affect survival of either HBMEC (A) or HIBCPP cells (B). Cells either pre-incubated with WitA for 0.5 h or non-treated were infected with wild type *Lm* EGD-e (MOI = 10) and co-incubated for 4 h. Subsequently, following removal of bacteria, cells were analyzed by life/dead assay and visualized and assessed using IF microscopy. Metabolically active cells are stained green, nuclei of dead cells are stained red. The presented images show fields of view representative for each of the observed samples across multiple experiments. A1, B1: control (untreated cells); A2-A5, B2-B5: infected cells (with *Lm* EGD-e wt); A3-A5, B3-B5: WitA-pre-incubated cells (A3, B3: 1 µM WitA; A4, B4: 5 µM WitA; A5, B5: 10 µM WitA).

A

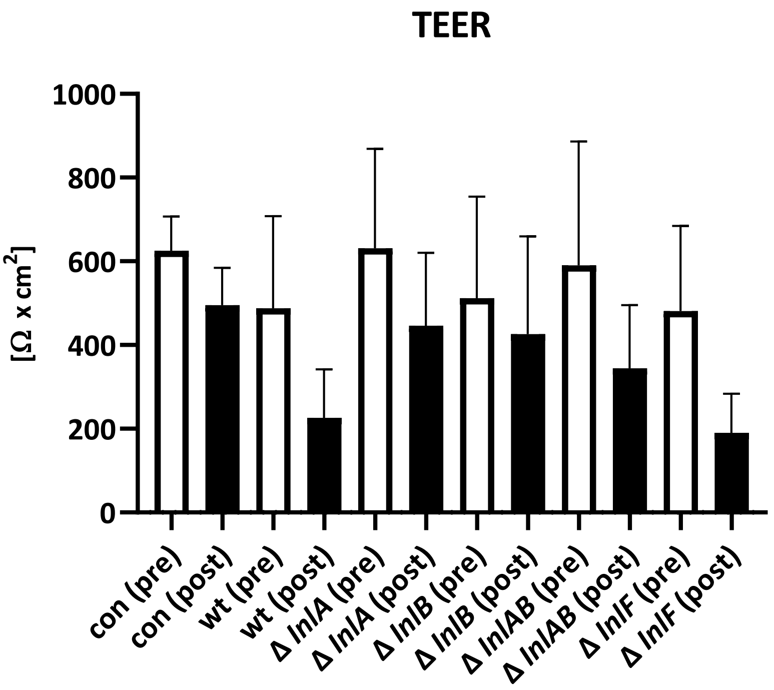

B

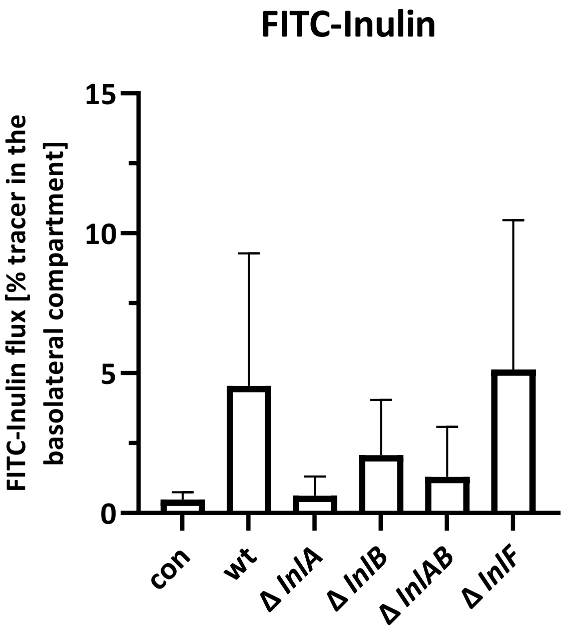

Supplementary Figure S3. HIBCPP cells grown in the inverted cell culture system were infected with wild type *Lm* EGD-e and the indicated mutants (MOI = 10) for 4 h or were left uninfected (con). (A) TEER values were measured at the beginning (pre) and the end (post) of the experiment. (B) The flux of FITC-labelled inulin (FITC-Inulin) was determined at the end of the experiment. Shown are the results of two independent experiments performed in triplicates.
